# Molecular Characterization of the Nsp2 and ORF5 (ORF5a) Genes of PRRSV Strains in Nine Provinces of China During 2016–2018

**DOI:** 10.3389/fvets.2021.605832

**Published:** 2021-03-04

**Authors:** Baishuang Yin, Shanshan Qi, Wanli Sha, Hongyu Qin, Liming Liu, Jinyan Yun, Jinhai Zhu, Guojiang Li, Dongbo Sun

**Affiliations:** ^1^College of Animal Science and Technology, Jilin Agricultural Science and Technology University, Jilin, China; ^2^Laboratory for the Prevention and Control of Swine Infectious Diseases, College of Animal Science and Veterinary Medicine, Heilongjiang Bayi Agricultural University, Daqing, China

**Keywords:** porcine reproductive and respiratory syndrome virus, Nsp2 gene, ORF5 gene, ORF5a gene, genetic evolution

## Abstract

Porcine reproductive and respiratory syndrome virus (PRRSV) causes a highly contagious disease and brings huge economic losses to commercial pork production worldwide. PRRSV causes severe reproductive failure in sows and respiratory distress in piglets. To trace the evolution of PRRSV in pigs with respiratory diseases in some regions of China, 112 samples were collected from nine provinces in China during 2016–2018. All samples were detected by RT-PCR and analyzed by the Nsp2/ORF5 (ORF5a)-genes-phylogeny. Sequence analysis and recombination analysis were conducted on the Nsp2/ORF5 (ORF5a) genes of the identified strain in the study. The RT-PCR result shown that the positive rate of PRRSV was 50.89% (57/112). Phylogenetic analysis showed that the identified PRRSV strains were all NA genotype and belonged to lineage 1, 3, and 8. The Nsp2 gene of identified PRRSV strains exhibited nucleotide homologies of 53.0 ~ 99.8%, and amino acid homologies of 46.8 ~ 99.7%. The ORF5 gene of identified PRRSV strains exhibited nucleotide homologies of 82.4 ~ 100%, and amino acid homologies of 79.6 ~ 100%. Sequence analysis revealed that a discontinuous 30-amino-acid deletion (positions 481 and 533–561) and a 131-amino-acid discontinuity deletion (positions 323–433, 481, and 533–551) in Nsp2 of PPRSV isolates; all identified strains in this study may be wild strains, and most identified strains may be highly virulent strains. Sequence analysis of ORF5 and ORF5a revealed that the mutation sites of GP5 were mainly concentrated in the signal peptide and epitopes region, while the mutation sites of ORF5a were mainly concentrated in the transmembrane and the intramembrane region. The recombination analysis indicated that there may be multiple recombination regions in identified strains, and the recombination pattern was more complex. This study showed that the prevalent PRRSV strain in some regions of China was still HP-PRRSV, while NADC30 strain also occupied a certain proportion; different types of PRRSV strains showed different patterns and variation in China. This study suggested that the monitoring of PRRSV prevalence and genetic variation should be further strengthened.

## Introduction

Porcine reproductive and respiratory syndrome (PRRS) is a major threat to the global swine industry, causing significant economic losses each year. The causative agent is PRRS virus (PRRSV), a member of the *Arteriviridae* family, order *Nidoviridales*. PRRSV is a single positive-strand RNA virus with a genome length of ~15.4 kb (1, 2). The PRRSV contains at least 10 open reading frames (ORFs), which are ORF1a, ORF1b, ORF2a, ORF2b, ORF5a, and ORF3 ~ 7 from the 5' to the 3' untranslated regions (UTR) (3, 4). ORF1a and ORF1b are cleaved into at least 13–16 non-structural proteins (Nsps) by a complex proteolytic cascade (3).

PRRSV was first reported in commercial pigs by the United States in 1987 (5), and the disease quickly spread worldwide with frequent break outs. PRRSV is still considered a highly contagious disease in the pig industry and creates huge economic losses (1, 6, 7). PRRSV was divided into two genotypes: the European genotype (type I) and North American genotype (type II) (3). There are three main subtypes of PRRSV (type II) isolates in Chinese pig populations: classical PRRSV (type II) including CH-1a, S1, and BJ-4; highly pathogenic PRRSV (HP-PRRSV) including JXA1, HuN4, and TJ; and NADC30-like PRRSV including JL580, CHsx1401, and HNjz15 (8). The genetic characteristic of HP-PRRSV isolates have a discontinuous 30-amino-acid deletion in Nsp2, and NADC30-like PRRSV isolates have a discontinuous 131-amino-acid deletion in Nsp2 (9, 10). PRRSV has mutated in the epidemic process to produce new strains due to the high frequency of gene mutation and recombination, in which new strains often have stronger environmental adaptations. The above factors have made the PRRSV epidemic more complicated, and it also brings great difficulties to disease prevention (11, 12). Nsp2 and ORF5 (ORF5a) are highly variable and ORF5 is associated with the neutralizing epitope (13, 14). They are usually used as target genes for PRRSV molecular epidemiological surveillance.

This study intends to reveal the prevalence and genetic evolution of PRRSV during 2016–2018 in different regions of China. The current study used the Nsp2 and ORF5 (ORF5a) genes to analyze the genetic evolution of the identified PRRSV strains. Our aim is to provide a theoretical basis for further monitoring of genetic variations of PRRSV in China.

## Methods

### Sampling

In total, 112 samples of the lung or lymph node tissues from pigs with respiratory diseases were collected between 2016 and 2018 in nine provinces or municipalities of China, including Heilongjiang, Jilin, Liaoning, Hubei, Jiangsu, Jiangxi, Zhejiang, Hebei, and the Inner Mongolia Autonomous Region. The lung or lymph node tissues were ground to powder with liquid nitrogen and diluted with three volumes of phosphate-buffered saline (PBS). The samples were centrifuged at 5,000 × g for 15 min at 4°C and the supernatants were transferred to a 1.5 mL tube. The genomic RNA was extracted from the supernatant using a commercial TIANamp Stool RNA Kit (Tiangen Biotech Co., Ltd, Beijing, China). The viral cDNA was synthesized using Moloney murine leukemia virus (RNaseH-) reverse transcriptase (Novoprotein Scientific Inc., Shanghai, China) in conjunction with six-random-nucleotide primers. The extracted genomic RNA and cDNA was stored at −80°C.

### PCR Detection and Sequencing of PRRSV Strains

The primer of ORF5 full-length gene (including complete ORF5a gene) can be found in the report by Cao et al. (15). A pair of primers of Nsp2 gene were designed based on the alignment of published PRRSV genome sequences obtained from the NCBI GenBank database. Primer information is shown in Table 1. The amplification reactions were carried out in a 25 μL reaction volume containing 12.5 μl of EmeraldAmp® PCR Master Mix (2×Premix) (TaKaRa Biotechnology Co., Ltd., Dalian, China), 0.5 μM of the forward primer, 0.5 μM of the reverse primer, 1 μL of cDNA, and an appropriate volume of double-distilled (dd) H_2_O. The cycling parameters of ORF5 gene were: 36 cycles of 94°C for 30 s, 55°C for 30 s, and 72°C for 1 min, followed by a final extension at 72°C for 10 min. The cycling parameters of Nsp2 gene were: 35 cycles of 95°C for 30 s, 57.6°C for 30 s, and 72°C for 3 min, followed by a final extension at 72°C for 10 min. The PCR products were analyzed by electrophoresis in a 1% agarose gel under UV light, and the samples with positive results were recorded. After the amplification, products were purified using the AxyPrep DNA Gel Extraction kit (A Corning Brand, Suzhou, China), and cloned into pGM-T Vector (TaKaRa Biotechnology Co., Ltd., Dalian, China). Each fragment was sequenced at least three times. All nucleotide sequences generated in this study have been submitted to the GenBank database.

**Table 1:** Primers specific for ORF5 and Nsp2 genes of PRRSV.

**Target**	**Primer**	**Primer sequence(5^**′**^-3^**′**^)**	**Amplified**
**gene**	**name**		**length(bp)**
ORF5/ORF5a	ORF5-F	GTTTTAGCCTGTCTTTTTGCC	731
	ORF5-R	TATATCATCACTGGCGTGTAGG	
Nsp2	Nsp2-nF	GAAGGGAATTGTGGTTGGCA	2 175 ~ 2 568
	Nsp2-nR	AGACCCAGAAAACACACCCA	

### Phylogenetic and Sequence Analysis of PRRSV Strains

For the phylogenetic analysis, the Nsp2 and ORF5 (ORF5a) genes of PRRSV reference strains were retrieved from the NCBI nucleotide database as reference sequences. Detailed information and the GenBank number of PRRSV reference strains is shown in Supplementary Table 1. To construct phylogenetic trees, nucleotide sequences of the target gene using the ClustalX alignment tool in the MEGA 6.06 software (16). Neighbor-joining phylogenetic trees were constructed with 1,000 bootstrap replicates and the remaining default parameters in the MEGA 6.06 software. The generated phylogenetic tree was annotated using the online software ITOL (https://itol.embl.de/) (17). The PRRSV-identified strains and reference strains were analyzed by MegAlign program in DNASTAR™ 5.06 software. Nucleotide/amino acid homology of Nsp2 and ORF5 (ORF5a) genes of PRRSV-identified strains, and reference strains were gained using the Pairwise/Multiple Align function in Geneious Prime software.

### Recombination Analysis

When RDP, GENECONV, BootScan, MaxChi, Chimera, SiScan, and 3eq methods were used to detect potential recombinant events, five or more methods were identified as gene recombination and *P* < 0.05 in RDP4.0 software. The strains in this event were determined to be recombinant strains. In addition, the detected recombination events were further confirmed by SimPlot 3.5.1.

## Results

### Detection and Analysis of PRRSV

In this study, 112 samples from nine provinces or municipalities of China were detected for PRRSV by RT-PCR. Of these tested samples, 50.89% (57/112) were positive for PRRSV. The infection rate of PRRSV was the highest in East China (85.71%, 12/14), including Jiangsu, Jiangxi, and Zhejiang province, and the lowest in Central China (11.11%, 1/9), including Hubei province. The infection rate of PRRSV in Northeast China was 52.3% (34/65), including Heilongjiang, Jilin, and Liaoning province, while the infection rate of PRRSV in North China was 43.5% (10/23), including Hebei province and the Inner Mongolia Autonomous Region (Table 2). Although the sources of the samples in different regions were non-uniform, the results showed that the presence of PRRSV was severe in some parts of China.

**Table 2:** The results of PRRSV sample positive rate in China between 2016 and 2018.

**Region**	**Province**	**Positive rate**	**Total**
Northeast China	Heilongjiang	60.46% (26/43)	52.3% (34/65)
	Jilin	0% (0/1)	
	Liaoning	36.36% (8/22)	
Central China	Hubei	11.11% (1/9)	11.11% (1/9)
East China	Jiangsu	100% (5/5)	85.7% (12/14)
	Jiangxi	100% (7/7)	
	Zhejiang	0% (0/2)	
North China	Inner Mongolia	44.44% (4/9)	43.5% (10/23)
	Hebei	42.86% (6/14)	
Total			50.89% (57/112)

### Phylogenetic Analysis of PRRSV

**Figure 1:**
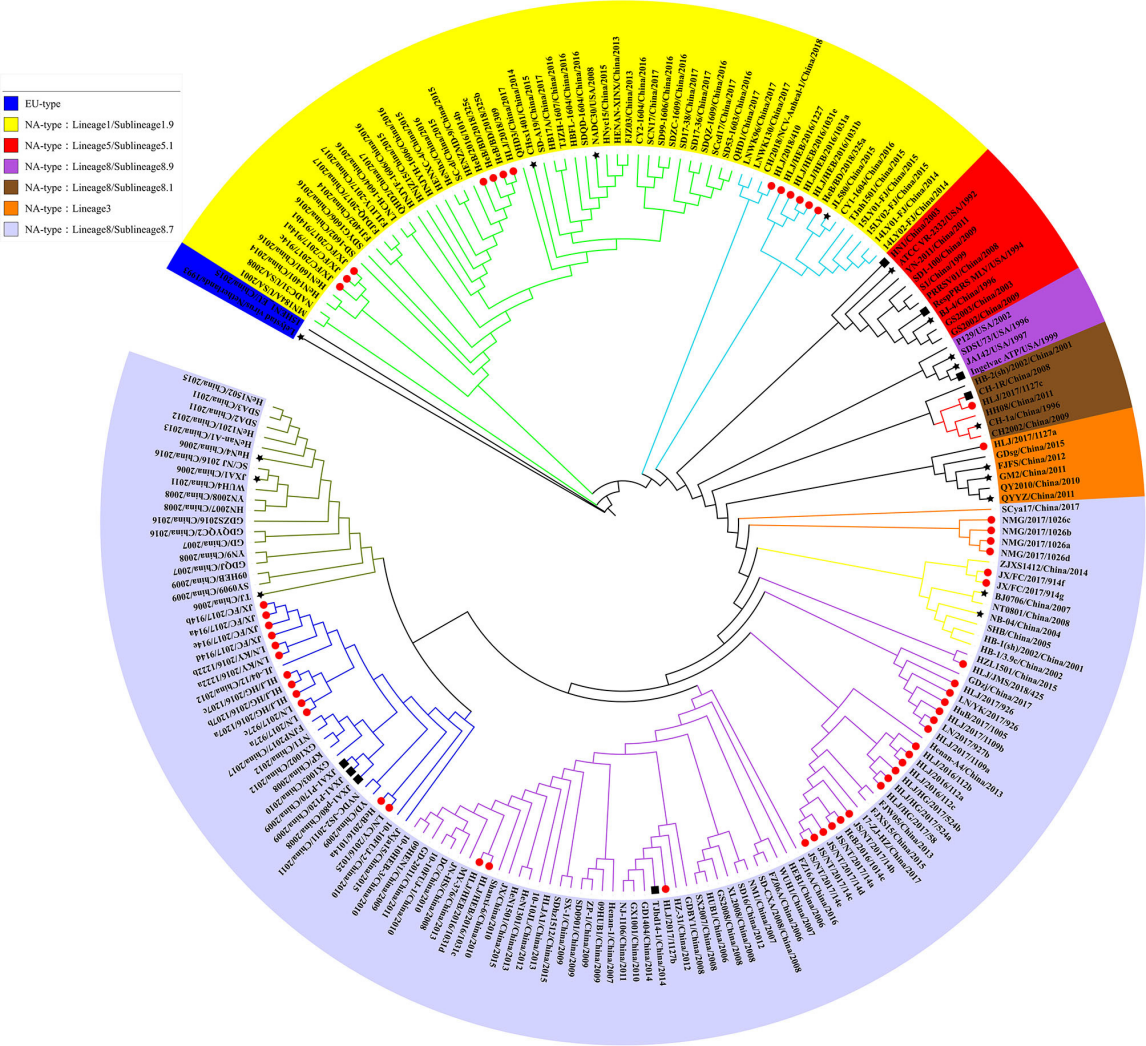
Phylogenetic analysis based on Nsp2 gene. The red circle diagram represents PRRSV strains identified in our study, black star diagram represents PRRSV reference strains, and the black square diagram represents PRRSV vaccine strains.

**Figure 2:**
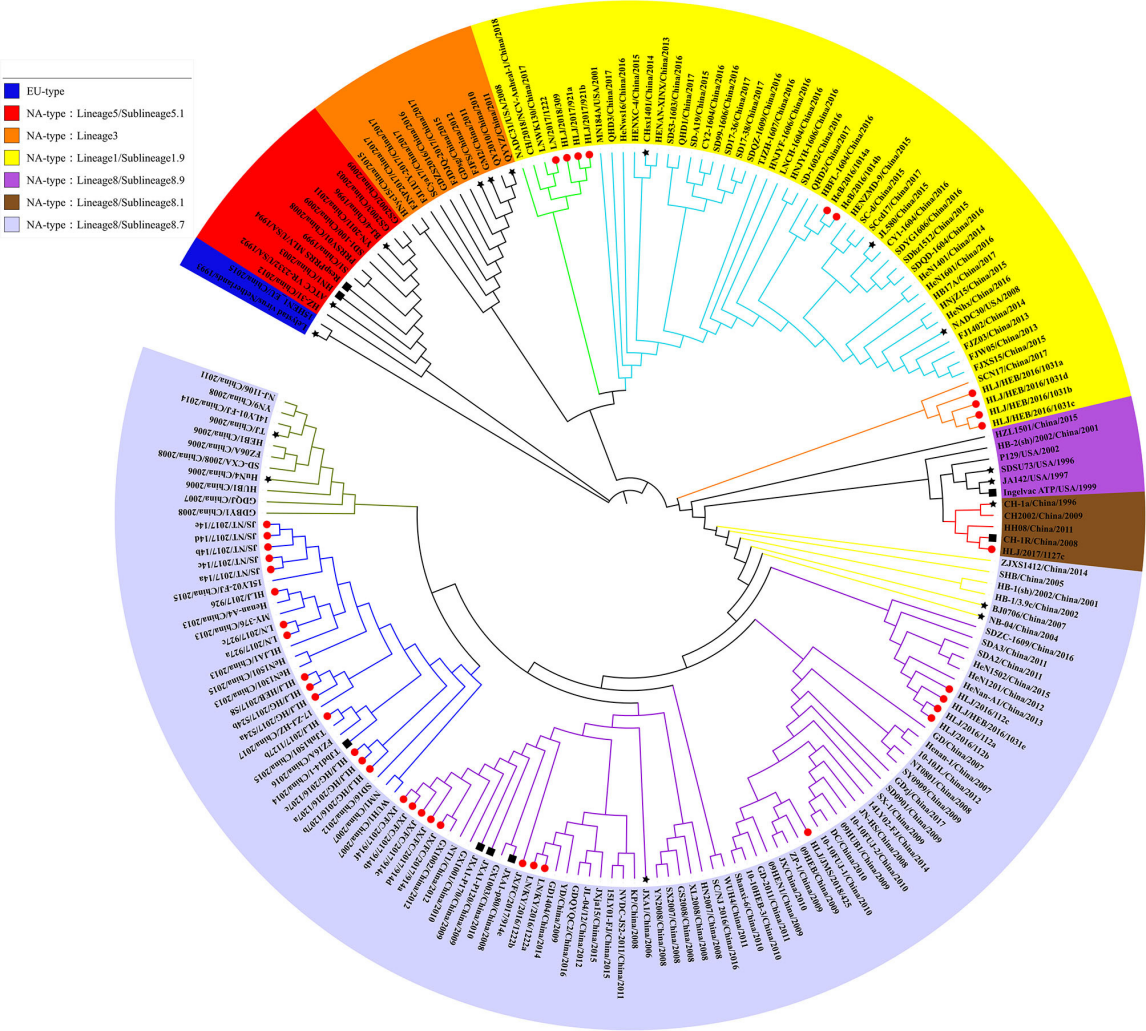
Phylogenetic analysis based on ORF5 gene. The red circle diagram represents PRRSV strains identified in our study, the black star diagram represents PRRSV reference strains, and the black square diagram represents PRRSV vaccine strains.

In 2010, Shi et al. conducted a systematic classification of type II PRRSVs based on ORF5 gene, and classified the PRRSVs into nine Lineage and 37 sublineage (18). Referring to the reported genotyping study by Shi et al., all the previously identified lineage reference sequences were also clustered in the same lineage in the phylogenetic tree of this study. The Nsp2 and ORF5-genes-based phylogenetic analysis revealed that all 56 PRRSV strains identified in this study belong to the North American genotype and were distributed in lineage 1, 3, and 8, among which the proportion of identified strains is the highest in sublineage 8.7 (73.2%, 41/56) and the lowest in lineage 3 (1.8%, 1/56) (Figures 1, 2). In addition, some identified strains were distributed differently in phylogenetic trees constructed with different genes. For instance, HeB/2016/1014a and HLJ/HEB/2016/1031(c, d) belong to the sublineage 8.7 while HLJ/HEB/2016/1031e and JX/FC/2017/914c belong to lineage 1 of the phylogenetic tree based on Nsp2 gene. However, the distribution of the identified strains was reversed in the phylogenetic tree constructed with the ORF5 gene. With the rapid growth of sequence deposition into the databases, it would be complicated for the diversity of PRRSV sequences.

### Sequence Analysis of PRRSV

#### Sequence Analysis of Nsp2 Genes of PRRSV

**Figure 3:**
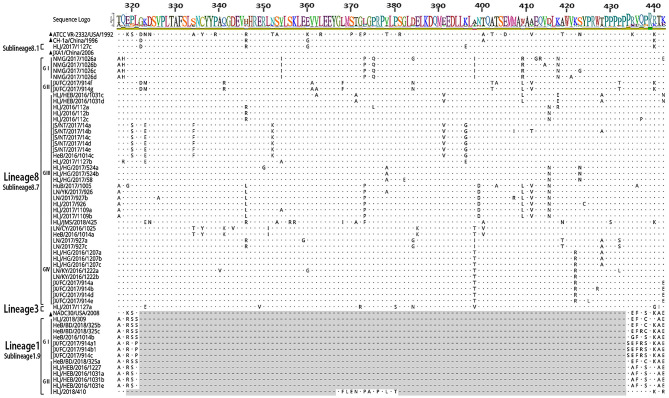
Amino acid sequence alignment of Nsp2 protein.

**Figure 4:**
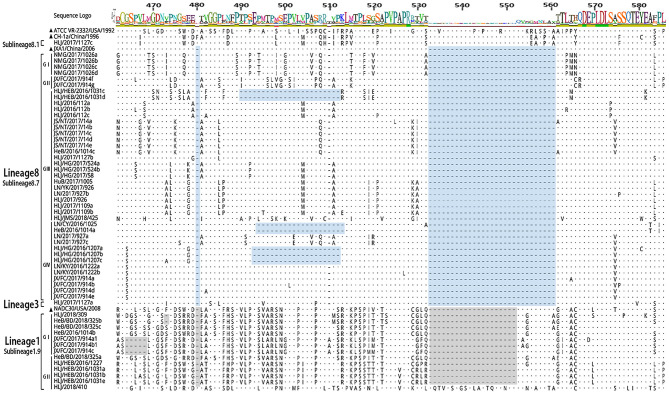
Amino acid sequence alignment of Nsp2 protein.

**Figure 5:**
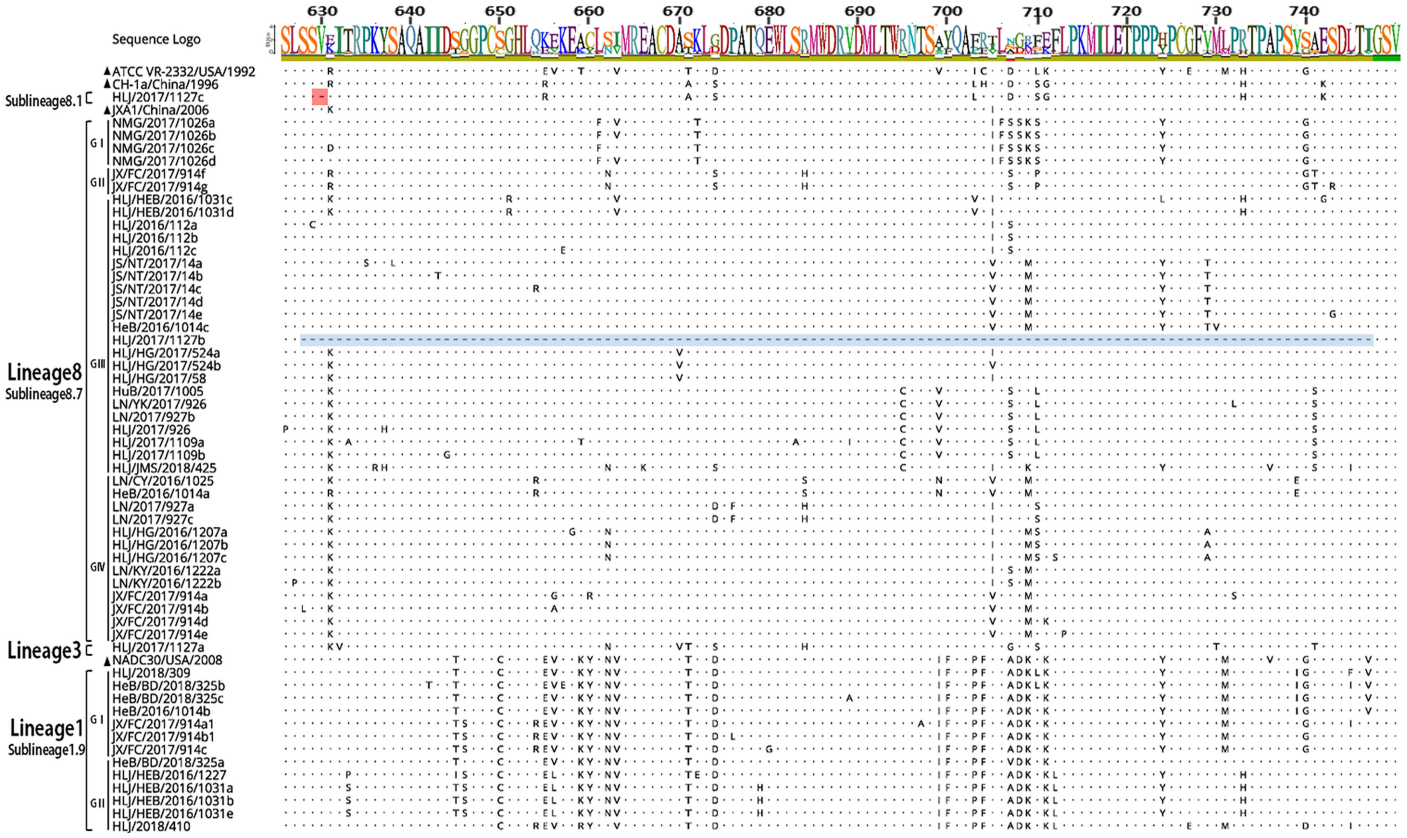
Amino acid sequence alignment of Nsp2 protein.

**Figure 6:**
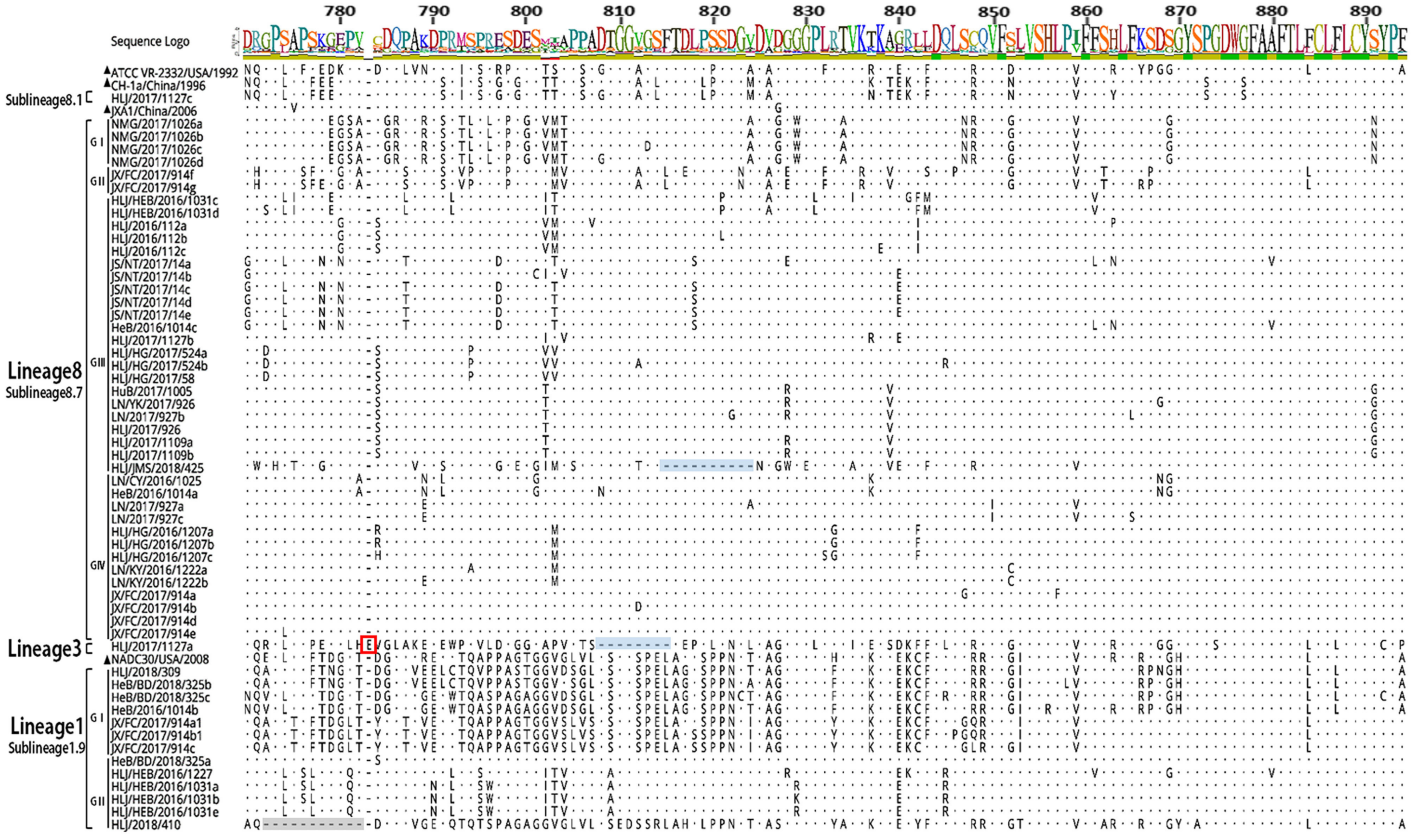
Amino acid sequence alignment of Nsp2 protein.

The 56 Nsp2 genes and 39 ORF5 (ORF5a) of PRRSV were successfully sequenced. The detailed GenBank number of the 56 PRRSV strains is shown in Supplementary Table 2. A sequence comparison of the Nsp2 genes revealed nucleotide homologies of 53.0 ~ 99.8% and deduced amino acid homologies of 46.8 ~ 99.7% among the 56 PRRSV strains. Along with the reference strain, its nucleotide and amino acid homologies compared with HP-PRRSV JXA1 strain was the highest (63.5 ~ 98.8% and 57.3 ~ 97.9%), and its nucleotide and amino acid homologies compared with NADC30 strain was the lowest (54.9 ~ 93.9% and 50.1 ~ 92.0%) (Table 3). In addition, the nucleotide and amino acid homologies of the identified strain in sublineage 8.1 and lineage 3 were higher than that of some identified strains in sublineage 8.7 from the same lineage. For example, HLJ/2017/1127c and HLJ/2017/1127b exhibited nucleotide and amino acid homologies of 76.6 and 73.1%, respectively. However, HLJ/2017/1127c and HLJ/2017/1127a exhibited nucleotide and amino acid homologies of 82.5 and 78.0%. The nucleotide and amino acid homologies of HLJ/2017/1127a and the representative strain QYYZ of lineage 3 was lower than the representative strain JXA1 of the 8.7 sublineage. This further suggests HLJ/2017/1127a is likely to be generated by recombinant strains in lineage 3 and sublineage 8.7. The classical PRRSV (type II) ATCC VR-2332 was used as the reference standard; the identified strain HLJ/2017/1127c had the same mutation pattern with vaccine strain CH-1R, which lacked a V at position 630 aa in the Nsp2. The identified strains of sublineage 8.7 and lineage 3 all showed a discontinuous 30-amino-acid deletion (positions 481 and 533–561) that conforms to the classical deletion mutation pattern of the HP-PRRSV-like strain. Excluding HLJ/2018/410, all strains identified in lineage 1 showed 131-amino-acid discontinuity deletion (positions 323–433, 481, and 533–551), that conforms to the classical deletion mutation pattern of the NADC30-like strain (Figures 3–6). The prevalent PRRSV strain in some regions of China was still HP-PRRSV, while NADC30 strain also occupied a certain proportion.

**Table 3:** Nucleotide and deduced amino acid homologies analysis based on Nsp2 gene (%).

		**Identified strain**				**Reference strain**					
		**Sublineage 8.1**	**Lineage 3**	**Lineage 1**	**Sublineage 8.7**	**VR-2332**	**CH-1a**	**JXA1**	**NADC30**	**QYYZ**	**JA142**
Sublineage 8.1	nt	100	82.5	62.3 ~ 69.7	76.6 ~ 88.5	85.9	98.8	89.0	65.2	81.9	92.6
	aa	100	78.0	55.3 ~ 63.7	73.1 ~ 84.3	81.7	98.4	85.1	59.7	76.2	89.0
Lineage 3	nt		100	61.9 ~ 67.1	77.3 ~ 88.4	76.1	82.6	88.5	63.9	74.7	79.6
	aa		100	55.9 ~ 62.0	73.8 ~ 84.7	70.6	77.8	85.7	58.7	70.5	74.3
Lineage 1	nt			72.5 ~ 99.6	**53.0**^**a**^ ~ 73.5	63.4 ~ 68.3	62.2 ~ 69.7	63.5 ~ 73.4	76.5 ~ 93.9	56.1 ~ 62.5	62.2 ~ 67.7
	aa			66.0 ~ 99.5	**46.8**^**a**^ ~ 69.3	56.8 ~ 62.9	55.3 ~ 63.7	57.3 ~ 69.3	72.0 ~ 92.0	49.1 ~ 55.6	55.9 ~ 61.7
Sublineage 8.7	nt				77.8 ~ **99.8**^**b**^	69.1 ~ 80.3	76.5 ~ 88.5	85.5 ~ 98.8	54.9 ~ 65.8	65.1 ~ 77.0	73.0 ~ 84.9
	aa				74.7 ~ **99.7**^**b**^	63.4 ~ 74.3	72.6 ~ 84.2	84.3 ~ 97.9	50.1 ~ 60.8	59.6 ~ 71.6	68.3 ~ 80.1
All strain	nt					63.4 ~ 85.9	62.2 ~ 98.8	63.5 ~ 98.8	54.9 ~ 93.9	56.1 ~ 81.9	62.2 ~ 92.6
	aa					56.8 ~ 81.7	55.3 ~ 98.4	57.3 ~ 97.9	50.1 ~ 92.0	49.1 ~ 76.2	55.9 ~ 89.0

*Mark a at the top right for the lowest value, Mark b at the top right for the highest value*.

#### Sequence Analysis of ORF5 Genes of PRRSV

**Figure 7:**
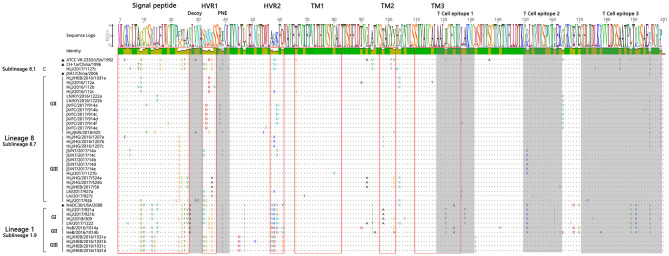
Amino acid sequence alignment of GP5 protein.

A sequence comparison of the ORF5 genes revealed nucleotide homologies of 82.4 ~ 100% and deduced amino acid homologies of 79.6 ~ 100% among the 39 PRRSV strains. The nucleotide and amino acid homologies compared with JXA1 strain was the highest (84.9 ~ 99.7%, 84.1 ~ 99.0%), and its nucleotide and amino acid homologies compared with QYYZ strain was the lowest (81.9 ~ 84.6%, 80.1 ~ 86.6%) (Table 4). The mutation sites of ORF5 were mainly concentrated in the signal peptide and epitopes region. GP5 virulence-related sites showed that nine of the 39 identified strains had mutated at the position 13th aa (R → Q). A total of 12 identified strains of sublineage 8.7 and lineage 1 had mutated at position 151 aa (R → K), The 137th aa of all identified strains was conservative and was S (Figure 7). It is observed that all identified strains may be wild strains, and most identified strains may be highly virulent strains in the study.

**Table 4:** Nucleotide and deduced amino acid homologies analysis based on ORF5 gene (%).

		**Identified strain**			**Reference strain**					
		**Sublineage 8.1**	**Sublineage 8.7**	**Lineage 1**	**VR-2332**	**CH-1a**	**JXA1**	**NADC30**	**QYYZ**	**JA142**
Sublineage 8.1	nt	100	90.9 ~ 94.0	84.9 ~ 91.0	91.0	98.7	94.4	87.1	84.4	93.4
	aa	100	87.1 ~ 89.6	81.1 ~ 88.6	89.1	97.5	90.5	85.1	81.6	94.0
Sublineage 8.7	nt		91.4 ~ **100**^**b**^	**82.4**^**a**^ ~ 92.2	86.4 ~ 89.2	91.9 ~ 95.0	94.9 ~ 99.7	83.1 ~ 86.1	81.9 ~ 84.4	88.1 ~ 91.0
	aa		91.0 ~ **100**^**b**^	**79.6**^**a**^ ~ 91.5	84.6 ~ 89.1	89.6 ~ 92.0	93.5 ~ 99.0	83.6 ~ 87.1	80.1 ~ 83.1	88.1 ~ 90.5
Lineage 1	nt			85.9 ~ **100**^**b**^	84.7 ~ 87.7	85.6 ~ 91.9	84.9 ~ 92.5	88.2 ~ 94.0	83.6 ~ 84.6	85.4 ~ 90.2
	aa			85.1 ~ **100**^**b**^	83.1 ~ 87.1	83.6 ~ 91.0	84.1 ~ 90.5	89.6 ~ 94.0	81.6 ~ 86.6	85.1 ~ 91.0
All strain	nt				84.7 ~ 91.0	85.6 ~ 98.7	84.9 ~ 99.7	83.1 ~ 94.0	81.9 ~ 84.6	85.4 ~ 93.4
	aa				83.1 ~ 89.1	83.6 ~ 97.5	84.1 ~ 99.0	83.6 ~ 94.0	80.1 ~ 86.6	85.1 ~ 94.0

*Mark a at the top right for the lowest value, Mark b at the top right for the highest value*.

#### Sequence Analysis of ORF5a Genes of PRRSV

**Figure 8:**
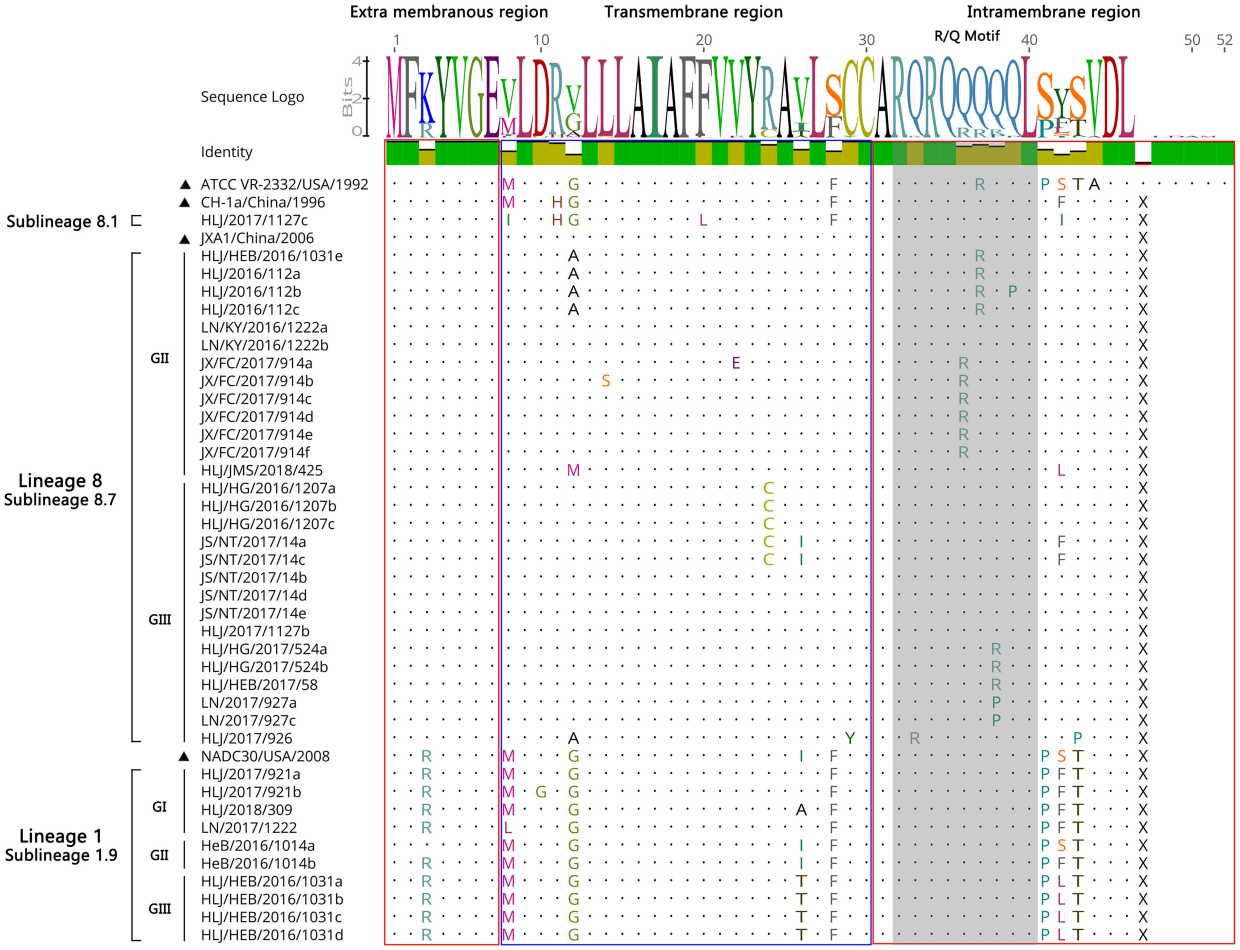
Amino acid sequence alignment of GP5a protein.

A sequence analysis of the ORF5a genes among the 39 PRRSV strains exhibited nucleotide homologies of 84.0 ~ 100% and deduced amino acid homologies of 80.8 ~ 100%. The nucleotide and amino acid homologies compared with JXA1 strain was the highest (87.2 ~ 99.4%, 84.6 ~ 100%), and its nucleotide and amino acid homologies compared with QYYZ strain was the lowest (70.5 ~ 79.5%, 67.3 ~ 78.8%) (Table 5). All the ORF5a proteins of the identified strains encoded 46 aa, and HLJ/2017/1127c exhibited high sequence similarity compared with CH-1a strain, with only a few point mutations, such as M^8^ → *I*^8^, F^20^ → *L*^20^, and S^42^/F^42^ → *I*^42^; A part of identified strains had two amino acid mutations in the transmembrane region, G^12^/V^12^ → *A*^12^, R^24^ → *C*^24^, and two amino acid mutations in the intramembrane region, Q^36^ → *R*^36^, Q^38^ → *R*^38^/P^38^. Compared with NADC30, some of the strains identified had one amino acid mutation in the transmembrane region, V^26^/I^26^ → *T*^26^, and one amino acid conformity mutation in the intramembrane region, S^42^ → *F*^42^/L^42^ (Figure 8). This shows that the mutation sites of ORF5a were mainly concentrated in the transmembrane region and the intramembrane region.

**Table 5:** Nucleotide and deduced amino acid homologies analysis based on ORF5a gene (%).

		**Identified strain**			**Reference strain**					
		**Sublineage 8.1**	**Sublineage 8.7**	**Lineage 1**	**VR-2332**	**CH-1a**	**JXA1**	**NADC30**	**QYYZ**	**JA142**
Sublineage 8.1	nt	100	87.8 ~ 90.4	87.2 ~ 90.4	80.1	97.4	91.0	88.5	74.4	83.3
	aa	100	82.7 ~ 88.5	84.6 ~ 86.5	75.0	94.2	88.5	84.6	69.2	80.8
Sublineage 8.7	nt		89.7 ~ **100**^**b**^	**84.0**^**a**^ ~ 91.0	76.9 ~ 81.4	89.7 ~ 92.9	93.6 ~ 99.4	83.3 ~ 88.5	70.5 ~ 74.4	76.9 ~ 80.8
	aa		86.5 ~ **100**^**b**^	**80.8**^**a**^ ~ 86.5	71.2 ~ 76.9	84.6 ~ 90.4	92.3 ~ 100	80.8 ~ 84.6	67.3 ~ 71.2	75.0 ~ 80.8
Lineage 1.9	nt			88.5 ~ **100**^**b**^	77.6 ~ 81.4	89.7 ~ 92.0	87.2 ~ 90.4	91.0 ~ 97.4	75.0 ~ 79.5	78.2 ~ 81.4
	aa			92.3 ~ **100**^**b**^	80.8 ~ 84.6	88.5 ~ 92.3	84.6 ~ 86.5	94.2 ~ 98.1	73.1 ~ 78.8	78.8 ~ 86.5
All strain	nt				76.9 ~ 81.4	89.7 ~ 97.4	87.2 ~ 99.4	83.3 ~ 97.4	70.5 ~ 79.5	76.9 ~ 83.3
	aa				71.2 ~ 84.6	84.6 ~ 94.2	84.6 ~ 100	80.8 ~ 98.1	67.3 ~ 78.8	75.0 ~ 86.5

*Mark a at the top right for the lowest value, Mark b at the top right for the highest value*.

### Recombination Analysis

The recombination analysis of Nsp2 gene showed that there were five potential recombination events (Table 6). The recombination analysis of Nsp2 gene showed that the recombinant strains in event 1 and 2 were produced by recombination of lineage 1 and sublineage 8.7 (Supplementary Figure 1). The high frequency mutation and recombination make the virus gain more genetically diverse (19). This recombination pattern is the most common in PRRSV recombinant strains in China, and animal tests have confirmed that the virulence of some recombinant strains is higher than the prototype strain NADC30 (9). The identified strain HLJ/2017/1127a in recombinant event 3 was produced by recombination of lineage 3 and sublineage 8.7 wild strain. The main parental strain was FZ06A, while the minor parental strain was QYYZ. Previous studies have shown that the low virulence prototype strain QYYZ even became highly virulent after recombination with the vaccine strain derived from HP-PRRSV (20). The identified strain JS/NT/2017/14b in recombinant event four showed that the main parental strain HLJ/2017/1127b belongs to subline 8.7, while the minor parental strain JS/NT/2017/14a belongs to subline 8.7. Three recombinant strains in recombinant event five were from the same origin as JS/NT/2017/14b, but the recombinant sites are different. The recombination analysis of ORF5 genes showed that the recombinant event included four recombinant strains of lineage 1 (Table 7). The main parental strain CY1-1604 belongs to lineage 1, while the minor parental strain GS2008 belongs to sublineage 8.7 (Supplementary Figure 2). Combined with the recombination analysis of Nsp2 gene, the identified strain HLJ/HEB/2016/1031 (a, b) was also recombined in ORF5 gene, which indicated that there may be multiple recombination regions in identified strains, and the recombination pattern was more complex.

**Table 6:** Recombination analysis of Nsp2 gene.

**Recombination event**	**Recombinant strains**	**Main parental strain**	**Minor parental strain**	**Recombinant breakpoint**	**Recombination analysis method**
1	HeB/2018/325a	HENZMD-9	10-10FUJ-2/China	18-2043	RDP (*P* = 1.22 × 10^−18^)
	HLJ/2016/1031a			(18–1591)	GENECONV (*P* = 9.49 × 10^−28^)
	HLJ/2016/1031b				BootScan (*P* = 1.78 × 10^−28^)
	HLJ/2016/1031e				MaxChi (*P* = 4.14 × 10^−26^)
	HLJ/HEB/1227				Chimaera (*P* = 6.68 × 10^−8^)
					SiScan (*P* = 9.44 × 10^−27^)
					3seq (*P* = 3.61 × 10^−8^)
2	JX/2017/914c	HENZMD-9	10-10FUJ-1/China	654–2600	RDP (*P* = 6.81 × 10^−40^)
	JX/2017/914a1			(654–2015)	GENECONV (*P* = 3.43 × 10^−34^)
	JX/2017/914b1				BootScan (*P* = 6.79 × 10^−28^)
					MaxChi (*P* = 7.71 × 10^−20^)
					Chimaera (*P* = 4.31 × 10^−22^)
					SiScan (*P* = 2.28 × 10^−25^)
					3seq (*P* = 2.27 × 10^−14^)
3	HLJ/2017/1127a	FZ06A	QYYZ	1892–2730	RDP (*P* = 2.08 × 10^−5^)
				(1742–2442)	GENECONV (NS)
					BootScan (*P* = 6.97 × 10^−5^)
					MaxChi (*P* = 6.41 × 10^−18^)
					Chimaera (*P* = 9.90 × 10^−16^)
					SiScan (*P* = 1.48 × 10^−8^)
					3seq (*P* = 7.66 × 10^−15^)
4	JS/NT/2017/14b	HLJ/2017/1127b	JS/NT/2017/14a	732–2227	RDP (*P* = 4.54 × 10^−7^)
				(732–2077)	GENECONV (*P* = 4.83 × 10^−6^)
					BootScan (*P* = 7.00 × 10^−8^)
					MaxChi (*P* = 2.44 × 10^−11^)
					Chimaera (NS)
					SiScan (*P* = 7.43 × 10^−12^)
					3seq (*P* = 3.88 × 10^−12^)
5	JS/NT/2017/14d	HLJ/2017/1127b	JS/NT/2017/14a	561–2534	RDP (*P* = 1.75 × 10^−5^)
	JS/NT/2017/14c			(561–2267)	GENECONV (*P* = 2.27 × 10^−4^)
	JS/NT/2017/14e				BootScan(*P* = 1.16 × 10^−5^)
					MaxChi (*P* = 6.46 × 10^−8^)
					Chimaera (NS)
					SiScan (*P* = 7.01 × 10^−17^)
					3seq (*P* = 8.06 × 10^−9^)

*NS, No significant P-value*.

**Table 7:** Recombination analysis of ORF5 gene.

**Recombination event**	**Recombinant strains**	**Main parental strain**	**Minor parental strain**	**Recombinant breakpoint**	**Recombination analysis method**
1	HLJ/HEB/2016/1031a	CY1-1604	GS2008	249–580	RDP (*P* = 1.92 × 10^−7^)
	HLJ/HEB/2016/1031b			(249–580)	GENECONV (*P* = 2.51 × 10^−6^)
	HLJ/HEB/2016/1031c				BootScan (*P* = 1.72 × 10^−6^)
	HLJ/HEB/2016/1031d				MaxChi (*P* = 2.60 × 10^−10^)
					Chimaera (*P* = 3.00 × 10^−9^)
					SiScan (*P* = 6.72 × 10^−12^)
					3seq (*P* = 2.29 × 10^−12^)

## Discussion

Since the outbreak of HP-PRRSV in 2006, PRRSV has been widely spread across the world. In previous studies, the positive rate of PRRSV was shown to be 55.21% (7,490/11,3567) in 29 provinces of China in 2012–2015 (21). In Central and Southern China, there was a positive rate of 50.62% (530/1,047) of PRRSV among 257 pig farms (22). In our study, the total positive rate of PRRSV was 50.89% (57/112) in nine provinces of China from 2016 to 2018, which was in accordance with the above scholars. PRRS is one of the most prevalent and threatening infectious diseases in Chinese pig farms.

Nsp2 and ORF5 (ORF5a) genes have the highest variability in PRRSV genome and are used as main target genes for PRRSV genetic variation. Phylogenetic tree analysis showed that all the 56 PRRSV strains identified in this study belong to the North American genotype and were distributed in lineage 1, 3, and 8 according to Shi et al. (18). In this study, the HP-PRRSV strain accounted for the highest proportion of epidemic strains in China; the NADC30-like strain had increased gradually, which was in accordance with the results of Gao et al. (23). However, some studies have shown that the NADC30-like strain in some regions of China have replaced the HP-PRRSV strain, and has become a new dominant strain (22). The rising infection rate of HP-PRRSV and NADC30-like strains may lead to a significant decrease in the effective protection rate of vaccines on pig farms.

The sequence alignments of Nsp2 gene revealed that the identified strain HLJ/2017/1127c in subline 8.1 had a high similarity with vaccine strain CH-1R, and existed a V deletion in the 630aa, suggesting that the identified strain may be a vaccine strain or a recombinant strain of a vaccine strain. Sequence alignments identified a discontinuous 30-amino-acid deletion (positions 481 and 533–561) and a 131-amino-acid discontinuity deletion (positions 323–433, 481, and 533–551) in Nsp2 of PPRSV isolates. JX/FC/2017/914(c, a1, b1) had the same deletion pattern as PRRSV strains HeN1401 and HeN1601, isolated by Zhang et al. (24). The recombinant analysis of the two epidemic strains revealed that HeN1401 and HeN1601 strains were generated by the recombinant weak vaccine strains TJbd14-1 and NADC30 (24). This further suggests that the identified strain JX/FC/2017/914(c, a1, b1) may also be a recombinant strain.

The GP5 protein sequences of different subline strains showed high similarity with the representative strains of the subline. The mutation sites of GP5 were mainly concentrated in the signal peptide and epitopes region. But some identified strains also have some amino acid consistent mutations in immune-related regions. Allende et al. found that nine amino acid site mutations may be closely related to the virulence of the PRRSV and that two sites (13 and 151 aa) were located in GP5 protein. The GP5 protein of high virulence strains generally were shown as R^13^ and R^151^ (25). Wesley et al. showed that the 137aa of GP5 protein can distinguish the attenuated vaccine strain (A^137^) and the wild strain (S^137^) (26). Therefore, the above three amino acid sites are often used to predict the virulence of PRRSV strains. The sequence analysis of GP5 protein showed that only one mutation pattern (R^13^ → *Q*^13^ and R^151^ → *K*^151^) existed in this study. Nine identified strains had mutated at position 13 aa, and 12 identified strains mutated at position 151 aa. In addition, the 137 aa of all identified strains is S. The results suggest that all identified strains may be wild strains, and most identified strains may be highly virulent strains in nine provinces of China during 2016–2018.

Studies have shown that ORF5a protein is essential for viral viability and infectivity (27, 28). There is fairly limited information available on current genetic variations of PRRSV ORF5a gene (29). Therefore, this study explored the genetic variation of ORF5a gene of PRRSV epidemic strains in China by molecular biological methods. The ORF5a protein generally encoded 46–51 amino acids of which ORF5a protein of PRRSV strains encoded 46 amino acids in lineage 1 and 8, and 51 amino acids in lineage 3 and 5 (30). All the ORF5a proteins identified in this study encoded 46 amino acids. Compared with the reference strain, the identified strains also showed high sequence similarity, and the mutation sites of ORF5a were mainly concentrated in the transmembrane region and the intramembrane region, while the other region was highly conserved. Our study demonstrated the existence of multiple different strains in the same region and extensive genetic mutation of PRRSV in China from 2016 to 2018.

The recombination analysis indicated that there may be multiple recombination regions in identified strains, and the recombination pattern was more complex. At present, many studies have shown that PRRSV strain in lineage 1 is prone to recombinant mutation, and some of the recombinant strains are more virulent than others (10, 19). Although the recombination pattern of the virus identified in this study is in accordance with that reported by some previous scholars, the change of pathogenicity of PRRSV by gene recombination is not absolute. This study only carried out partial gene (Nsp2 and ORF5) recombination analysis without the virus isolation and whole genome sequences of PRRSV, so the recombination of whole genome sequences is more complicated and different.

## Conclusion

This study showed that PRRSV infection was prevalent in nine provinces of China from 2016 to 2018, and the prevalent PRRSV strain in most regions was still HP-PRRSV, while the NADC30 strain also occupied a certain proportion. There was a discontinuous 30-amino-acid deletion (positions 481 and 533–561) and a 131-amino-acid discontinuity deletion (positions 323–433, 481, and 533–551) in Nsp2 of PPRSV isolates. All identified strains in this study may be wild strains, and most identified strains may be highly virulent strains. This study identified highly homologous HP-PRRSV variants with distinct genetic mutation, which contributes to further analyzing the epidemics and evolution of PRRSV in the field.

## Data Availability Statement

The datasets presented in this study can be found in online repositories. The names of the repository/repositories and accession number(s) can be found in the article/Supplementary Material.

## Author Contributions

DS and GL conceived the study. BY, SQ, and JZ analyzed the data. BY and SQ wrote the manuscript for submission. WS, HQ, LL, and JY participated in the design of the study, performed the data collection and analysis, and commented on the manuscript. All authors approved the final manuscript.

## Conflict of Interest

The authors declare that the research was conducted in the absence of any commercial or financial relationships that could be construed as a potential conflict of interest.
