# Supplementary material for: Molecular Characterization of the Nsp2 and ORF5 (ORF5a) Genes of PRRSV Strains in Nine Provinces of China During 2016–2018
Source: Front Vet Sci. 2021 Mar 4;8:605832. doi: 10.3389/fvets.2021.605832 (PMC7969665; doi:10.3389/fvets.2021.605832)
Supplement: Supplementary file 1 [file Data_Sheet_1.docx]

**TABLE S1 Information of PRRSV reference strains**

| No. | Strain name | Origin | GenBank no. | No. | Strain name | Origin | GenBank no. |
| --- | --- | --- | --- | --- | --- | --- | --- |
| 1 | Lelystad virus | Netherlands, 1993 | M96262 | 44 | PRRSV01 | China, 2008 | FJ175687 |
| 2 | ATCC VR-2332 | USA, 1992 | U87392 | 45 | KP | China, 2008 | GU232735 |
| 3 | RespPRRS MLV | USA, 1994 | AF066183 | 46 | YN9 | China, 2008 | GU232738 |
| 4 | SDSU73 | USA, 1996 | JN654458 | 47 | JN-HS | China, 2008 | HM016158 |
| 5 | JA142 | USA, 1997 | AY424271 | 48 | GDBY1 | China, 2008 | GQ374442 |
| 6 | Ingelvac ATP | USA, 1999 | DQ988080 | 49 | ZP-1 | China, 2009 | HM016159 |
| 7 | MN184A | USA, 2001 | DQ176019 | 50 | SD1-100 | China, 2009 | GQ914997 |
| 8 | P129 | USA, 2002 | AF494042 | 51 | GS2002 | China, 2009 | EU880441 |
| 9 | NADC30 | USA, 2008 | JN654459 | 52 | CH2002 | China, 2009 | EU880438 |
| 10 | NADC31 | USA, 2008 | JN660150 | 53 | YD | China, 2009 | JF748717 |
| 11 | CH-1a | China, 1996 | AY032626 | 54 | SX-1 | China, 2009 | GQ857656 |
| 12 | CH-1R | China, 2008 | EU807840 | 55 | SY0909 | China, 2009 | HQ315837 |
| 13 | BJ-4 | China, 1996 | AF331831 | 56 | 09HEB | China, 2009 | JF268679 |
| 14 | S1 | China, 1999 | DQ459471 | 57 | 09HEN1 | China, 2009 | JF268684 |
| 15 | HB-1(sh)/2002 | China, 2001 | AY150312 | 58 | 09HUB1 | China, 2009 | JF268682 |
| 16 | HB-2(sh)/2002 | China, 2001 | AY262352 | 59 | SD0901 | China, 2009 | JN256115 |
| 17 | HB-1/3.9c | China, 2002 | HQ233605 | 60 | DC | China, 2010 | JF748718 |
| 18 | GS2003 | China, 2003 | EU880442 | 61 | GX1003 | China, 2010 | JX912249 |
| 19 | HN1 | China, 2003 | AY457635 | 62 | QY2010 | China, 2010 | JQ743666 |
| 20 | NB-04 | China, 2004 | FJ536165 | 63 | 10-10HEB-3 | China, 2010 | JQ663553 |
| 21 | SHB | China, 2005 | EU864232 | 64 | Shanxi-6 | China, 2010 | KJ855518 |
| 22 | JXA1 | China, 2006 | EF112445 | 65 | GX1001 | China, 2010 | JQ955657 |
| 23 | JXA1-p80 | China, 2008 | FJ548853 | 66 | 10-10FUJ-2 | China, 2010 | JQ663547 |
| 24 | JXA1-P120 | China, 2009 | KC422727 | 67 | 10-10FUJ-1 | China, 2010 | JQ663546 |
| 25 | JXA1-P170 | China, 2009 | JQ804986 | 68 | JX | China, 2010 | JX317649 |
| 26 | HuN4 | China, 2006 | EF635006 | 69 | GM2 | China, 2011 | JN662424 |
| 27 | TJ | China, 2006 | EU860248 | 70 | QYYZ | China, 2011 | JQ308798 |
| 28 | HEB1 | China, 2006 | EF112447 | 71 | GD-2011 | China, 2011 | KC527830 |
| 29 | HUB1 | China, 2006 | EF075945 | 72 | NJ-1106 | China, 2011 | JX880029 |
| 30 | FZ06A | China, 2006 | MF370557 | 73 | NVDC-JS2-2011 | China, 2011 | JQ715698 |
| 31 | BJ0706 | China, 2007 | GQ351601 | 74 | HH08 | China, 2011 | JX679179 |
| 32 | GD | China, 2007 | EU825724 | 75 | SDA3 | China, 2011 | JX878380 |
| 33 | WUH1 | China, 2007 | EU187484 | 76 | SDA2 | China, 2011 | JX878379 |
| 34 | Henan-1 | China, 2007 | EU200962 | 77 | WUH4 | China, 2011 | JQ326271 |
| 35 | NM1 | China, 2007 | EU860249 | 78 | YN-2011 | China, 2011 | JX857698 |
| 36 | GDQJ | China, 2007 | GQ374441 | 79 | HZ-31 | China, 2012 | KC445138 |
| 37 | NT0801 | China, 2008 | HQ315836 | 80 | GX1002 | China, 2012 | JQ955658 |
| 38 | HN2007 | China, 2008 | EU880437 | 81 | SD16 | China, 2012 | JX087437 |
| 39 | SX2007 | China, 2008 | EU880434 | 82 | 10-10JL | China, 2012 | JQ663554 |
| 40 | SD-CXA/2008 | China, 2008 | GQ359108 | 83 | JL-04/12 | China, 2012 | JX177644 |
| 41 | XL2008 | China, 2008 | EU880436 | 84 | FJFS | China, 2012 | KP998476 |
| 42 | YN2008 | China, 2008 | EU880435 | 85 | NT1 | China, 2012 | KP179402 |
| 43 | GS2008 | China, 2008 | EU880431 | 86 | HeN1201 | China, 2012 | MF689000 |
| 87 | MY-376 | China, 2013 | KJ609517 | 123 | GDQYQC2 | China, 2016 | MF526896 |
| 88 | Henan-A4 | China, 2013 | KJ534539 | 124 | SDYG1606 | China, 2016 | KY053458 |
| 89 | HeNan-A1 | China, 2013 | KJ002451 | 125 | HeN1601 | China, 2016 | MF766474 |
| 90 | HENAN-XINX | China, 2013 | KF611905 | 126 | SC/NJ 2016 | China, 2016 | MF818049 |
| 91 | HLJA1 | China, 2013 | KT351739 | 127 | CY1-1604 | China, 2016 | MH651736 |
| 92 | FJZ03 | China, 2013 | KP860909 | 128 | CY2-1604 | China, 2016 | MH651737 |
| 93 | FJW05 | China, 2013 | KP860911 | 129 | HNJYF-1606 | China, 2016 | MH651738 |
| 94 | HeN1301 | China, 2013 | MF766470 | 130 | HBFL-1604 | China, 2016 | MH651739 |
| 95 | GD1404 | China, 2014 | MF669720 | 131 | HNJYH-1606 | China, 2016 | MH651740 |
| 96 | ZJXS1412 | China, 2014 | MF669722 | 132 | LNCH-1604 | China, 2016 | MH651741 |
| 97 | HeN1401 | China, 2014 | MF766471 | 133 | SDQD-1604 | China, 2016 | MH651742 |
| 98 | FJ1402 | China, 2014 | KX169191 | 134 | SD-1602 | China, 2016 | MH651743 |
| 99 | CHsx1401 | China, 2014 | KP861625 | 135 | SD53-1603 | China, 2016 | MH651744 |
| 100 | 14LY01-FJ | China, 2014 | KP780881 | 136 | SD99-1606 | China, 2016 | MH651745 |
| 101 | 14LY02-FJ | China, 2014 | KP780882 | 137 | SDQZ-1609 | China, 2016 | MH651746 |
| 102 | TJbd14-1 | China, 2014 | KP742986 | 138 | SDZC-1609 | China, 2016 | MH651747 |
| 103 | GDsg | China, 2015 | KX621003 | 139 | TJZH-1607 | China, 2016 | MH651748 |
| 104 | FJXS15 | China, 2015 | KX758250 | 140 | GDZS2016 | China, 2016 | MH046843 |
| 105 | TJnh1501 | China, 2015 | KX510269 | 141 | LNWK96 | China, 2017 | MG860516 |
| 106 | HNjZ15 | China, 2015 | KT945017 | 142 | SCcd17 | China, 2017 | MG914067 |
| 107 | HNyc15 | China, 2015 | KT945018 | 143 | FJDJQ-2017 | China, 2017 | MG011719 |
| 108 | JL580 | China, 2015 | KR706343 | 144 | FJLIUY-2017 | China, 2017 | MG011718 |
| 109 | HENXC-4 | China, 2015 | KU950371 | 145 | HB17A | China, 2017 | MG844181 |
| 110 | JXja15 | China, 2015 | KR149645 | 146 | GDzj | China, 2017 | MF772778 |
| 111 | 15LY01-FJ | China, 2015 | KU215416 | 147 | SD17-36 | China, 2017 | MH121061 |
| 112 | 15LY02-FJ | China, 2015 | KU215417 | 148 | 17-ZJ-HZ | China, 2017 | MF770574 |
| 113 | SD-A19 | China, 2015 | MF375260 | 149 | SD17-38 | China, 2017 | MH068878 |
| 114 | SC-d | China, 2015 | MF375261 | 150 | SCN17 | China, 2017 | MH078490 |
| 115 | SDhz1512 | China, 2015 | KX980392 | 151 | SCya17 | China, 2017 | MH324400 |
| 116 | HZL1501 | China, 2015 | MF669721 | 152 | LNWK130 | China, 2017 | MG913987 |
| 117 | HeN1501 | China, 2015 | MF766472 | 153 | QHD2 | China, 2017 | MH167387 |
| 118 | HeN1502 | China, 2015 | MF766473 | 154 | QHD3 | China, 2017 | MH167388 |
| 119 | 15HEN1_EU | China, 2015 | KX967492 | 155 | QHD1 | China, 2017 | MG687491 |
| 120 | HENZMD-9 | China, 2015 | KU950374 | 156 | FJNP2017 | China, 2017 | MH046842 |
| 121 | FZ16A | China, 2016 | KY761966 | 157 | CH/2018/NCV-Anheal-1 | China, 2018 | MH370474 |
| 122 | HeNhx | China, 2016 | KX766379 |  |  |  |  |

**TABLE S2 Information of identified PRRSV Strains**

| Identified strain name | GenBank no. | | |
| --- | --- | --- | --- |
|  | Nsp2 gene | ORF5 gene | ORF5a gene |
| HLJ/HEB/2016/1031a | MH422011 | MH422059 | MK341743 |
| HLJ/HEB/2016/1031b | MH422012 | MH422060 | MK341744 |
| HLJ/HEB/2016/1031c | MH422013 | MH422061 | MK341745 |
| HLJ/HEB/2016/1031d | MH422014 | MH422062 | MK341746 |
| HLJ/HEB/2016/1031e | MH422015 | MH422063 | MK341747 |
| HLJ/2016/112a | MH422016 | MH422064 | MK341748 |
| HLJ/2016/112b | MH422017 | MH422065 | MK341749 |
| HLJ/2016/112c | MH422018 | MH422066 | MK341750 |
| LN/CY/2016/1025 | MH422019 | — | — |
| HeB/2016/1014a | MH422020 | MH422067 | MK341751 |
| HeB/2016/1014b | MH422021 | MH422068 | MK341752 |
| HeB/2016/1014c | MH422022 | — | — |
| LN/KY/2016/1222a | MH422023 | MH422069 | MK341753 |
| LN/KY/2016/1222b | MH422024 | MH422070 | MK341754 |
| HLJ/HG/2016/1207a | MH422025 | MH422071 | MK341755 |
| HLJ/HG/2016/1207b | MH422026 | MH422072 | MK341756 |
| HLJ/HG/2016/1207c | MH422027 | MH422073 | MK341757 |
| JS/NT/2017/14a | MH422028 | MH422074 | MK341758 |
| JS/NT/2017/14b | MH422029 | MH422075 | MK341759 |
| JS/NT/2017/14c | MH422030 | MH422076 | MK341760 |
| JS/NT/2017/14d | MH422031 | MH422077 | MK341761 |
| JS/NT/2017/14e | MH422032 | MH422078 | MK341762 |
| HLJ/HEB/2016/1227 | MH422033 | — | — |
| HLJ/HG/2017/524a | MH422034 | MH422079 | MK341763 |
| HLJ/HG/2017/524b | MK334127 | MH422080 | MK341764 |
| HLJ/HG/2017/58 | MK334128 | MH422081 | MK341765 |
| HLJ/2017/1127a | MH422037 | — | — |
| HLJ/2017/1127b | MH422038 | MH422082 | MK341766 |
| HLJ/2017/1127c | MH422039 | MH422083 | MK341767 |
| HuB/2017/1005 | MH422040 | — | — |
| LN/YK/2017/926 | MH422041 | — | — |
| LN/2017/927a | MH422042 | MH422086 | MK341770 |
| LN/2017/927b | MH422043 | — | — |
| LN/2017/927c | MH422044 | MH422087 | MK341771 |
| NMG/2017/1026a | MH422045 | — | — |
| NMG/2017/1026b | MH422046 | — | — |
| NMG/2017/1026c | MH422047 | — | — |
| NMG/2017/1026d | MH422048 | — | — |
| JX/FC/2017/914a | MH422049 | MH422088 | MK341772 |
| JX/FC/2017/914a1 | MK334125 | — | — |
| JX/FC/2017/914b | MH422050 | MH422089 | MK341773 |
| JX/FC/2017/914b1 | MK334126 | — | — |
| JX/FC/2017/914c | MH422051 | MH422090 | MK341774 |
| JX/FC/2017/914d | MH422052 | MH422091 | MK341775 |
| JX/FC/2017/914e | MH422053 | MH422092 | MK341776 |
| JX/FC/2017/914f | MH422054 | MH422093 | MK341777 |
| JX/FC/2017/914g | MH422055 | — | — |
| HLJ/2017/926 | MH422056 | MH422094 | MK341778 |
| HLJ/2017/1109a | MH422057 | — | — |
| HLJ/2017/1109b | MH422058 | — | — |
| HLJ/2017/921a | — | MH422084 | MK341768 |
| HLJ/2017/921b | — | MH422085 | MK341769 |
| LN/2017/1222 | — | MK192128 | MK341780 |
| HeB/BD/2018/325a | MK192121 | — | — |
| HeB/BD/2018/325b | MK192122 | — | — |
| HeB/BD/2018/325c | MK192123 | — | — |
| HLJ/2018/309 | MK192124 | MK192127 | MK341779 |
| HLJ/2018/410 | MK192125 | — | — |
| HLJ/JMS/2018/425 | MK192126 | MK192129 | MK341781 |


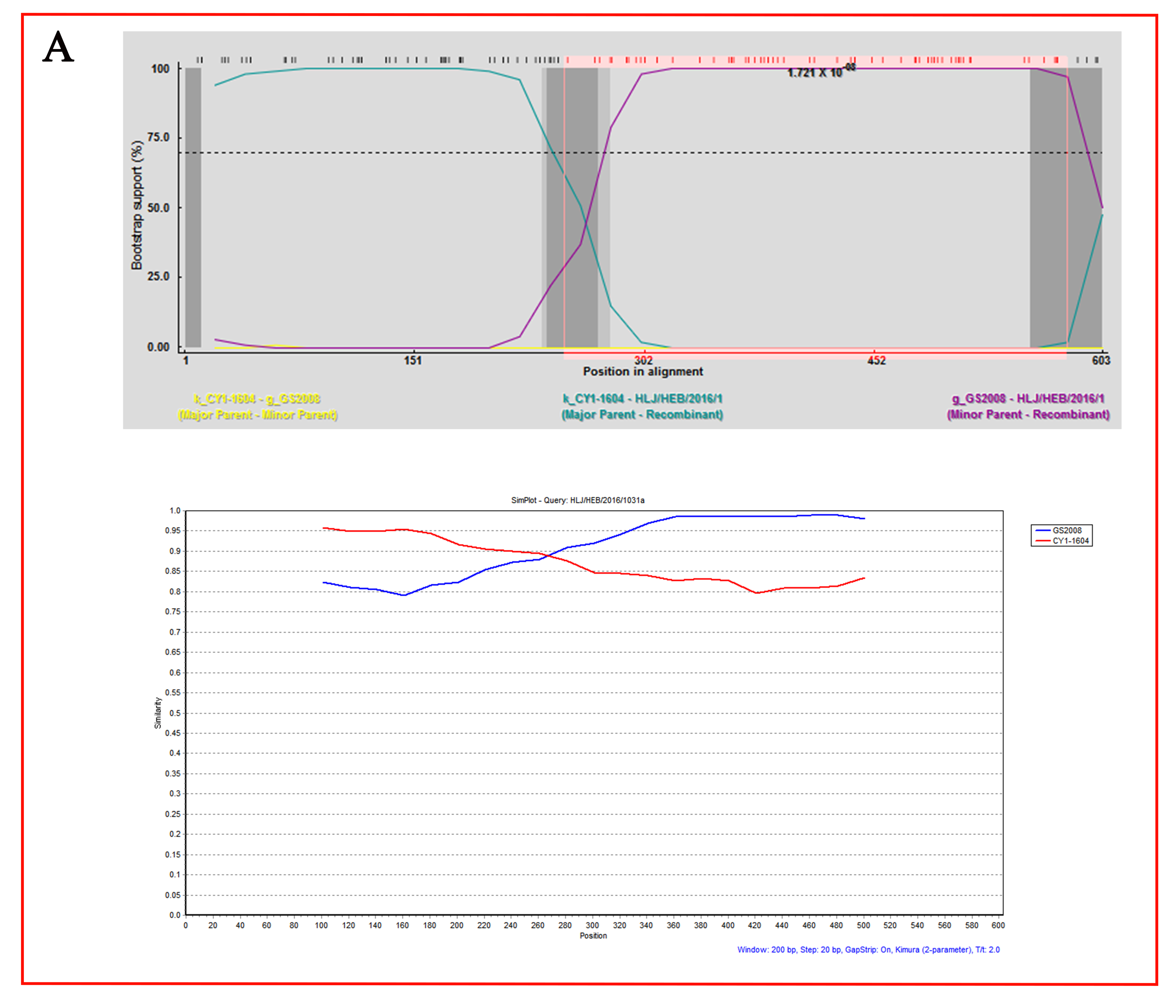


**FIGURE S2 BootScan and Simplot verification of ORF5 gene recombination event**


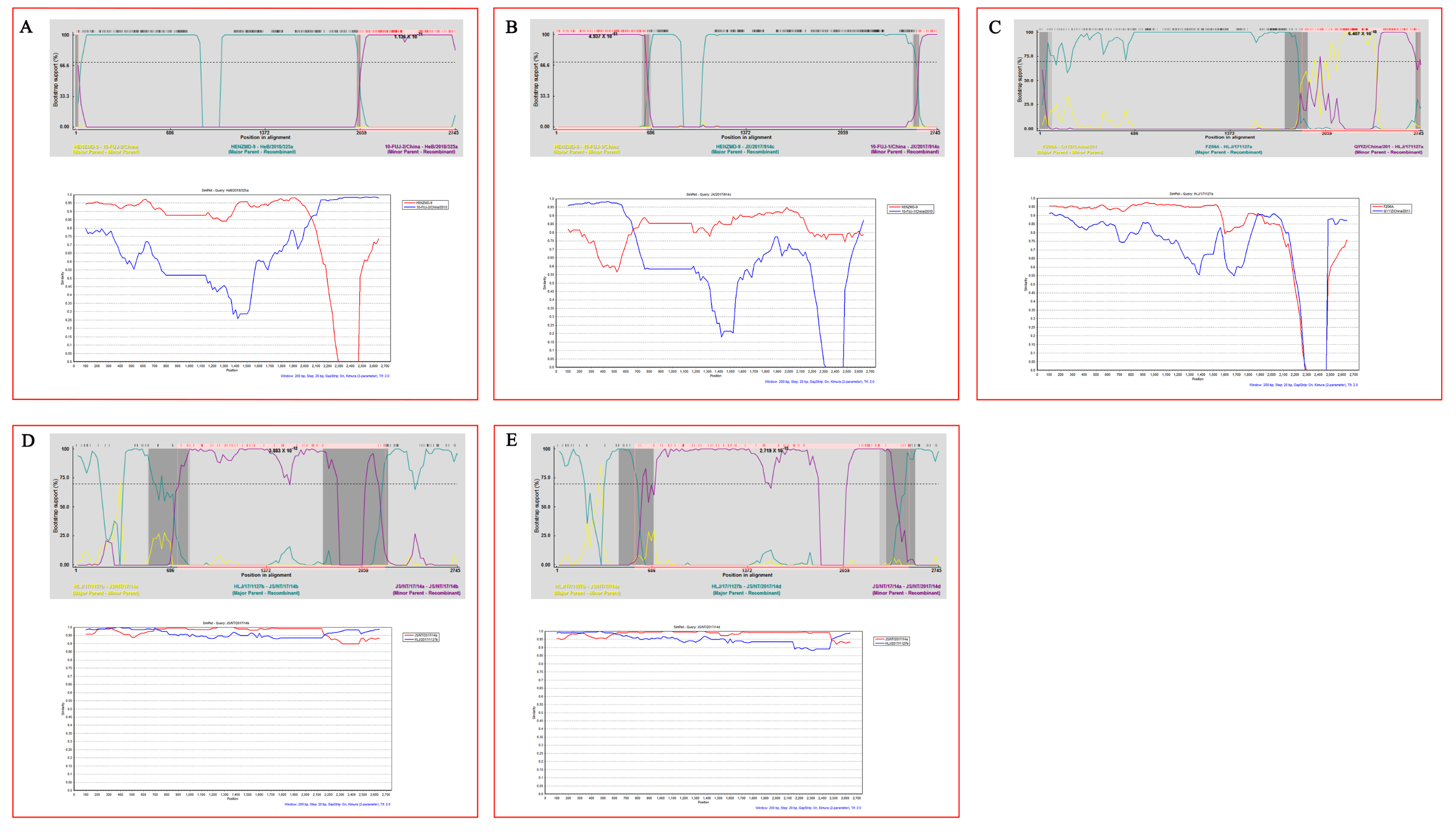


**FIGURE S1 BootScan and Simplot verification of Nsp2 gene recombination event**
